# Intelligent electromagnetic metasurface camera: system design and experimental results

**DOI:** 10.1515/nanoph-2021-0665

**Published:** 2022-01-04

**Authors:** Zhuo Wang, Hongrui Zhang, Hanting Zhao, Tie Jun Cui, Lianlin Li

**Affiliations:** Department of Electronics, State Key Laboratory of Advanced Optical Communication Systems and Networks, Peking University, Beijing 100871, China; State Key Laboratory of Millimeter Waves, Southeast University, Nanjing 210096, China; Pazhou Laboratory, Guangzhou 510330, China

**Keywords:** deep learning, electromagnetic sensing, programmable metasurfaces

## Abstract

Electromagnetic (EM) sensing is uniquely positioned among nondestructive examination options, which enables us to see clearly targets, even when they visually invisible, and thus has found many valuable applications in science, engineering and military. However, it is suffering from increasingly critical challenges from energy consumption, cost, efficiency, portability, etc., with the rapidly growing demands for the high-quality sensing with three-dimensional high-frame-rate schemes. To address these difficulties, we propose the concept of intelligent EM metasurface camera by the synergetic exploitation of inexpensive programmable metasurfaces with modern machine learning techniques, and establish a Bayesian inference framework for it. Such EM camera introduces the intelligence over the entire sensing chain of data acquisition and processing, and exhibits good performance in terms of the image quality and efficiency, even when it is deployed in highly noisy environment. Selected experimental results in real-world settings are provided to demonstrate that the developed EM metasurface camera enables us to see clearly human behaviors behind a 60 cm-thickness reinforced concrete wall with the frame rate in order of tens of Hz. We expect that the presented strategy could have considerable impacts on sensing and beyond, and open up a promising route toward smart community and beyond.

## 1 Introduction

Nowadays, electromagnetic (EM) sensing is a powerful nondestructive examination tool under all-weather all-time operational condition, severing as a fundamental asset in science, engineering and military [1], [2], [3], [4], [5], [6], [7], [8], [9], [10], [11], [12], [13]. Three kinds of sensing strategies, i.e., synthetic aperture [2], real aperture [10] and coding aperture [7], have been developed by now. Typically, an entire sensing chain consists of two major constituting parts: data acquisition and data processing; however, their cost performance indexes using developed sensing strategies have to be traded-off. For instance, the coding-aperture strategy is capable of producing high-quality images with one or a few sensors, which is adorable in terms of hardware cost, but at the cost of computationally inefficient reconstruction algorithms [7], [8], [9]. In contrast, the real-aperture strategy has nearly no requirements on data processing, but requires costly massive sensors [10], [11], [12], [13]. This situation becomes more and more serious with the ever-increasing demand for the high-frame-rate three-dimensional imaging, since it is inevitably companied with dramatically increasing data rates that poses a heavy burden on the data acquisition, system communication and subsequent reconstruction algorithms. Furthermore, being constrained by the resourced-limited infrastructure space available or point-of-care sensing, sensors are needed to be as compact as possible. Thus, it is urgently demanded to develop cost-effective sensing schemes in favor of adaptive data acquisition and instant data processing.

In order to address aforementioned difficulties, we propose the intelligent EM metasurface camera by deploying inexpensive programmable metasurfaces [14], [15], [16] with artificial neural networks [17], [18], [19], [20]. From the perspective of data acquisition, the proposed EM metasurface camera works similar to compressive imagers [7], [8], [9]. Particularly, the programmable metasurface acts as an electronically-controllable coding aperture, which is used to manipulate dynamically the wavefield on physical level such that as much relevant target’s information as possible can be captured by one or a few fixed sensors in a compressive manner. As a matter of fact, the programmable metasurface, owing to its unique capability in manipulating flexibly EM wavefronts, has been intensively investigated for sensing and others over past years [21], [22], [23], [24], [25], [26], [27], [28]. Being different from them, to our best knowledge, this is the first effort with the programmable metasurface to realize the high-frame-rate imaging in real-world settings. Additionally, in this work, the entire EM sensing chain works in the learnable manner in the context of Bayesian learning principle, as opposed to that of Ref. [24] with the learned data processing alone. We here mean by the real-world setting that the target is in a really complicated indoor physical environment, and acquired signals are seriously disturbed by unknown co-channel interferences. In addition, another important contribution in this work is that a Bayesian inference framework for the developed EM metasurface camera has been proposed. Guided by this principle, our EM camera can work in an intelligence way, in the sense that it can be trained such that the measurements can be adaptively collected on physical level, and that the target can be recognized on digital level. As such, the high-fidelity video of the target can be obtained by using the developed intelligent EM metasurface camera.

In this article, we build a proof-of-principle prototype system of intelligent EM metasurface camera working at around 2.4 GHz and establish a Bayesian principle for it. We demonstrate experimentally that such EM metasurface camera is capable of enabling us to see clearly human behaviors behind a 60 cm-thickness reinforced concrete wall with high frame rate. We here would like to say that the frame rate can be achieved in order of tens of KHz in principle if more specialized transceivers are used rather than commercial radio devices (i.e., USRP). The presented sensing strategy could open up a promising route toward smart community and beyond, and can be readily transposed to other frequencies and other types of wave phenomena.

## 2 System design of intelligent EM metasurface camera

**Figure 1:**
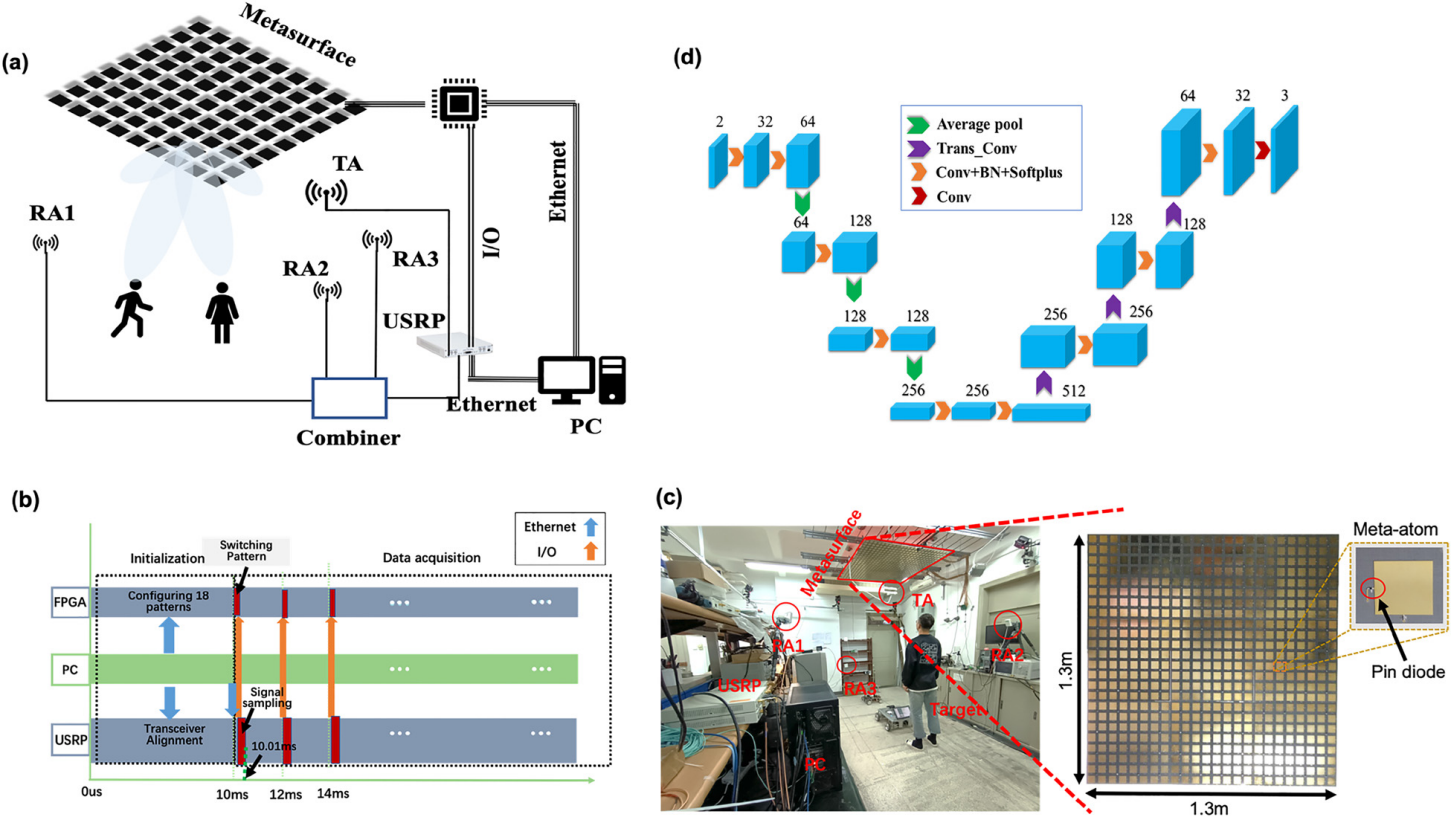
System configuration of the proposed intelligent EM metasurface camera working at around 2.4 GHz. (a) The sketch map of proposed intelligent EM metasurface camera system, which is composed of a large-aperture programmable metasurface, a USRP X310, a transmitting antenna (TA), a three-antenna (RA1, RA2, RA3) receiver and a personal computer (PC). (b) The operational procedure of data acquisition of the proposed EM camera, where a 10 ms-length system initialization procedure is marked before data acquisition. In addition, 2 ms-duration will be taken per control pattern of the programmable metasurface due to the USRP’s inherent limitation, through only 0.01 ms is needed for data acquisition per control pattern. (c) The experimental setup in indoor environment, where the photo of the reflection-type one-bit coding programmable metasurface with size of 1.3 m × 1.3 m has been provided as well. (d) The proposed U-net network along with necessary parameters.

The proposed intelligent EM metasurface camera is a software-defined system in favor of the high-frame-rate EM sensing. For purpose of principle illustration, the intelligent EM metasurface camera is designed to work at around commodity Wi-Fi frequency of 2.4 GHz, and is used for monitoring human behaviors in indoor environment. With reference to Figure 1a, the proposed EM metasurface camera consists of a large-aperture programmable metasurface, a low-cost commercial software-defined radio device (Ettus USRP X310), a transmitting antenna, a three-antenna receiver and a personal computer. Both the USRP and metasurface are communicated with the host computer via the Ethernet under the transmission control protocol (TCP); meanwhile, the USRP has I/O series communication with the metasurface. The host computer is responsible of calculating the control patterns and sends these patterns to the metasurface through FPGA module; at the same time, it sends a command signal to the USRP for synchronizing its transmitting and receiving channels, as shown in Figure 1b. Note that this initialization process takes about 10 ms. To trade-off the imaging quality with efficiency, we explore 18 patterns for compressive microwave measurement per image in this work. For each control pattern, the USRP under control of the host computer will generate the radio signal with chirp waveform, radiate it into the investigation domain through the transmitting antenna, and receive the echoes reflected from the target. It takes 0.01 ms to complete this whole measurement procedure; however, it costs 2 ms due to the USRP’s inherent limitation in our experiments. Thus, it will take 2 ms × 18 = 36 ms to produce a microwave image, implying the frame rate achievable is about 27 Hz. We here would like to say that if the USRP is updated with more specialized transceiver devices, the frame rate achievable can be optimized to be in order of tens of kHz in principle, and we leave this investigation in future. Afterward, the acquired echoes are processed by artificial neural networks in the host computer, which is directly responsible for the object reconstruction and recognition. In this work, the chip signal waveform transmitted by the USRP reads:

(1) $$s\left(t\right)=\text{exp}\left(j\left(2\pi f_{\text{c}}t+\pi Kt^{2}\right)\right),\;0\leq t\leq T$$

where $j=\sqrt{-1}$ , $f_{\text{c}}=2.424$ GHz is the carrier frequency, K=B/T denotes the sweep rate of the chirp, *B* = 50 MHz is the frequency bandwidth, and *T* = 10 μs is the Chirp pulse duration.

Programmable metasurface is a kind of engineered ultrathin material, which consists of a 2D array of controllable meta-atoms. By now, it has been intensively explored in many areas, e.g., sensing [21], [22], [23], [24], [25], [26], [27], [28], wireless communication [29, 30], analog computation [31, 32], wireless energy deposition [33], and so on. Here, the programmable metasurface controlled with artificial neural networks is utilized for two major purposes: (i) manipulating adaptively the EM wavefields toward the target, suppressing the unwanted disturbances from surrounding environment like walls, furnishings, and so on. (ii) Serving as an electronically-controllable coding aperture in compressive-sensing manner. Being different from the coding patterns explored in conventional compressive sensing strategies, the deep-learning-driven metasurface considered here is capable of generating the measurements which are consistent with those needed by the digital reconstruction, as detailed next section. In our implementation, the whole programmable metasurface is designed to be composed of 3 × 3 identical metasurface panels, and each panel has 8 × 8 meta-atoms with size of 54 × 54 mm^2^ (see Figure 1c). Each meta-atom soldiered with one PIN diode has binary distinct EM response states. Specifically, the reflection phase response changes by 180° around 2.4 GHz when the PIN diode is switched from OFF (ON) to ON (OFF), while the amplitudes remain almost unaltered. The statuses of PIN diodes are controlled by an FPGA-based micro-control-unit with clock of 50 MHz. The power needed to program the metasurface is minimal and can be as low as a few μ W per meta-atom. More details about the utilized metasurface can be found in our previous publications [24, 27, 28]. Owing to the large-view field nature enabled by the large-aperture programmable metasurface, the target’s information can be readily captured by two fixed receivers. Therefore, we expect that the reconstruction of target’s information can be readily achieved from the compressive measurements.

## 3 Bayesian principle for intelligent EM metasurface camera

Here, we will elaborate on the Bayesian principle for the data acquisition and processing behind the proposed intelligent EM metasurface camera. The Bayesian principle says that a self-organizing system that is at equilibrium with its supporting environment must minimize its free energy [34]. For our problem, assuming the target’s state $s_{t}$ at time *t*, the intelligent EM metasurface camera aims at organizing the measurement strategy $\pi_{t}$ , collecting measurements $o_{t}$ , and retrieving the target. It is noted that the measurements depend on the EM manipulation via the programmable metasurface, and thus the efficient measurement strategy can be achieved by changing the control coding pattern of the metasurface. To build a machine for this problem, we explore the probabilistic generative model and its Bayesian inference solution. For the target-sensor scenario with the generative distribution $P_{\varphi }\left(s_{\leq t},o_{\leq t}|\pi_{\leq t}\right)$ , the intelligent EM metasurface camera has a picture of it, which is characterized with a posterior distribution (i.e., recognition function) $Q_{\theta }\left(s_{\leq t}|o_{\leq t},\pi_{\leq t}\right)$ . Here, φ and θ encapsulate all trainable parameters defining $P_{\varphi }$ and $Q_{\theta }$ , respectively. Then, the generative network $P_{\varphi }$ and inference network $Q_{\theta }$ could be achieved by minimizing the following free energy [33], i.e.,

(2) $$ℱ=E_{Q_{\theta }\left(s_{\leq t}|o_{\leq t},\pi_{\leq t}\right)}\left[\text{ln}\left(\frac{Q_{\theta }\left(s_{\leq t}|o_{\leq t},\pi_{\leq t}\right)}{P_{\varphi }\left(s_{\leq t},o_{\leq t}|\pi_{\leq t}\right)}\right)\right]$$

Under well-known Markov chain approximation, Eq. (2) can be expressed as:

(3) $$ℱ=\overset{T}{\underset{t=1}{\sum }}J_{t}$$

where

$$\begin{array}{l} \;J_{t}=J_{t-1}-E_{Q_{\theta }\left(s_{\leq t-1}|o_{\leq t-1},\pi_{\leq t-1}\right)}\left[\underset{\text{Likelihood}}{\underbrace{E_{Q_{\theta }\left(s_{t}|o_{t},s_{\leq t-1},\pi_{t}\right)}\text{ln}\left(P_{\varphi }\left(o_{t}|s_{t},\pi_{t}\right)\right)}}\right]\\ \;-E_{Q_{\theta }\left(s_{\leq t-1}|o_{\leq t-1},\pi_{\leq t-1}\right)}\left[\underset{\text{KL divergence}}{\underbrace{E_{Q_{\theta }\left(s_{t}|o_{t},s_{\leq t-1},\pi_{t}\right)}\text{ln}\left(\frac{P_{\varphi }\left(s_{t}|s_{\leq t-1}\right)}{Q_{\theta }\left(s_{t}|o_{t},s_{\leq t-1},\pi_{t}\right)}\right)}}\right]. \end{array}$$

Note that $E_{Q_{\theta }\left(s_{t}|o_{t},s_{\leq t-1},\pi_{t}\right)}\text{ln}\left(P_{\varphi }\left(o_{t}|s_{t},\pi_{t}\right)\right)$ describes the likelihood or observation accuracy at time *t*, while $E_{Q_{\theta }\left(s_{t}|o_{t},s_{\leq t-1},\pi_{t}\right)}\text{ln}\left(\frac{P_{\varphi }\left(s_{t}|s_{\leq t-1}\right)}{Q_{\theta }\left(s_{t}|o_{t},s_{\leq t-1},\pi_{t}\right)}\right)$ , Kullback–Leibler (KL) distance or relative entropy, reflects the recognition complexity of $Q_{\theta }\left(s_{t}|o_{t},s_{\leq t-1},\pi_{t}\right)$ at time *t*. In order to facilitate numerical implementations, we here make several assumptions as follows:

(i)
$Q_{\theta }\left(s_{t}|o_{t},s_{\leq t-1},\pi_{t}\right)=N\left(s_{t}|f_{\theta }\left(o_{t},\pi_{t}\right),\alpha^{2}I\right)N\left(s_{t}|s_{t-1},\beta^{2}I\right)$ , where the nonlinear function $f_{\theta }$ is modeled with a U-net artificial neural network [35] (see Figure 1d), $\alpha^{2}$ and $\beta^{2}$ are two trainable parameters.
(ii)
$P_{\varphi }\left(s_{t}|s_{\leq t-1}\right)$ is modeled with so-called Brown motion [6, 20];
(iii)
$P_{\varphi }\left(o_{t}|s_{t},\pi_{t}\right)$ is represented with a physical-model-based neural network, i.e., $P_{\varphi }\left(o_{t}|s_{t},\pi_{t}\right)=N\left(o_{t}|A_{\pi_{t}}s_{t},\gamma^{2}I\right)$ , where $A_{\pi_{t}}$ is a linear operator defined through Eq. (4), and $\gamma^{2}$ is a trainable parameter.

Now, one can determines the measurement strategy $\pi_{t}$ , and generative network $P_{\varphi }$ and inference network $Q_{\theta }$ by minimizing Eq. (3) by exploring variational autoencoder method [27].

Before closing this section, we give more details about the U-net network modeling $f_{\theta }$ , as shown in Figure 1d. This proposed U-net network has double-channel input: one channel is from the real-part of preprocessed microwave signal, the other is from the imaginary part. Features of microwave signals are extracted layer by layer, and then gradually approach to the labeled IUV three-channel images [38]. For each layer of U-net, the residual network structure is adopted to prevent from so-called vanishing gradient, meanwhile speed up training procedure. Each residual network module is composed of three convolutional network layers, where a SoftPlus nonlinear activation operation and batch normalization (BN) follow after each convolutional layer. The training is performed over a GPU computer with a single Nvidia GTX2080Ti, and the training setup is made: the optimizer is Adam [36], learning rate is 10^−3^, weight decay rate is 5 × 10^−5^ and batch-size is 128. Although the whole investigation domain is really big, the target only occupies a small fraction of it. Taking this observation into account, we propose to enforce the values of the pixels outside the target to be zero each iteration during the training procedure, which is referred to as window Adam for notation convenience. By using this simple scheme, the training convergence can be considerably improved.

## 4 Experimental results

### 4.1 Data preprocessing

Our intelligent metasurface camera is deployed in a real-world indoor environment, leading to the seriously noisy measurements. Such EM camera works at around 2.4 GHz, thus the acquired signals are inevitably disturbed by unwanted but unknown in-band wireless signals (like, Wi-Fi, Bluetooth, etc.) everywhere. Moreover, there are a plenty of unwanted interferences arising from surrounding environment such as walls, furniture, and so on. Unfortunately, these disturbances are remarkably dominant over the acquired signals carrying the target’s information, and more importantly, the in-band inferences from commodity wireless signals are usually statistically non-stationary. Therefore, to get the acceptable imaging results, we have to build signal models and develop denoise methods for the developed metasurface camera.

Assuming that a transmitter at $r_{T}$ gives rise to a frequency-domain signal s(ω) , and a point-like object with reflection coefficient $\sigma \left(r_{o}\right)$ is situated at $r_{o}$ , where ω denotes the angular frequency. We here would like to say that such point-like target model makes sense and following discussions can be readily extended for the case of extended objects in terms of linear supposition principle in context of Born scattering approximation [1, 6]. Then, the echo acquired by the receiver at $r_{R}$ can be approximated as:

(4) $$\begin{array}{l} y\left(r_{R};\omega ,C\right)\approx s\left(\omega \right)\sigma \left(r_{o}\right)g\left(r_{R},r_{o};\omega \right)g\left(r_{T},r_{o};\omega \right)+s\left(\omega \right)\sigma \left(r_{o}\right)g\left(r_{R},r_{o};\omega \right)\left(\underset{n}{\sum }\Gamma_{n}^{C}\left(\omega \right)g\left(r_{T},r_{n};\omega \right)g\left(r_{o},r_{n};\omega \right)\right)\;+s\left(\omega \right)g\left(r_{R},r_{T};\omega \right)+s\left(\omega \right)\underset{n}{\sum }\Gamma_{n}^{C}\left(\omega \right)g\left(r_{R},r_{n};\omega \right)g\left(r_{T},r_{n};\omega \right)+\epsilon \left(\omega \right) \end{array}$$

Herein, $g\left(r_{R},r_{o};\omega \right)$ denotes the so-called Green’s function of considered physical environment, which characterize the system response at $r_{R}$ given a radio source at $r_{o}$ . $\Gamma_{n}^{C}\left(\omega \right)$ represents the reflection coefficient of the *n*th meta-atom at $r_{n}$ , when the metasurface is configured with the control coding pattern C . Note that the summation is performed over the metasurface meta-atoms. Moreover, ϵ(ω) accounts for disturbances from aforementioned in-band inferences, environment clutters, system noise, and others. In Eq. (4), other possible multiple-scattering terms have been ignored due to the deployment of the directional transmitting and receiving antennas. Note that the first and second terms in the right hand of Eq. (4) carry the target’s information; while other terms are usually target-independent. It is trivial to remove the third and fourth terms by exploring a simple background removal operation; however, to filter out the last term is challenging due to its statistically non-stationary nature for the real-time application demand, since conventional filter-based methods, such as, the time-frequency filtering, principal component filtering, and others, are typically computationally prohibitive. In order to resolve this problem, the deep learning strategy is explored here. To this end, we design an end-to-end deep convolutional network, termed as Filter-CNN, which will map the noisy signal after background removal to desired denoised signal. Its training is performed in a GPU personal computer with Nvidia GTX2080Ti, and major parameters are set as follows: the optimizer is ADAM, batch-size = 128, initial learning rate = 0.01, and iteration epochs = 100. Such training costs 2 h. Once the Filter-CNN is trained well, the filtering time for a group of 20 × 1000 data is about 0.3 s, while conventional methods need at least 11 s.

**Figure 2:**
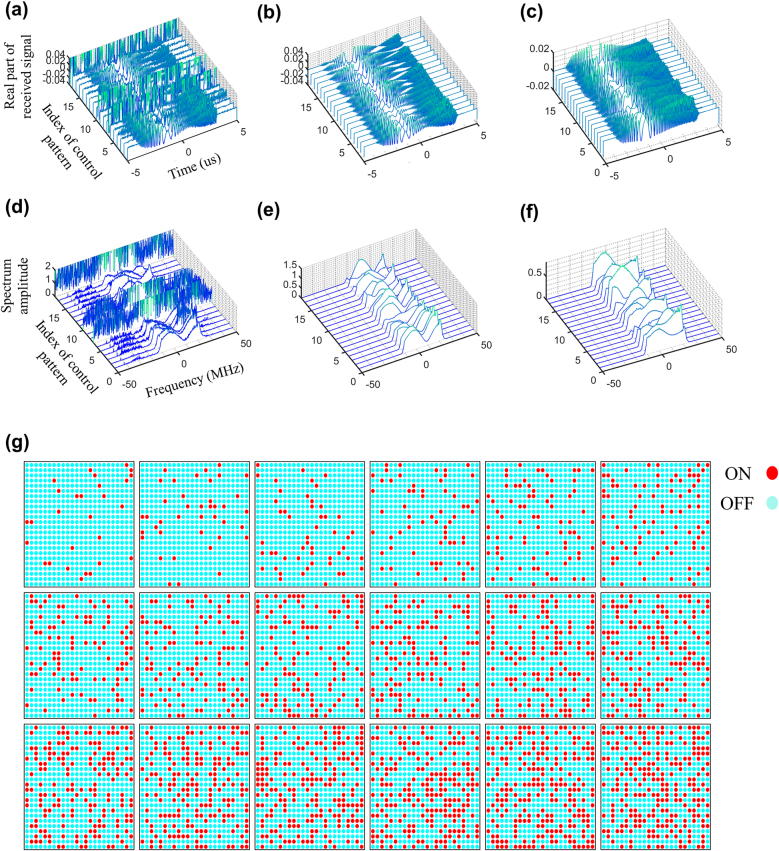
Experimental results of the signal denoise for proposed intelligent EM metasurface camera working at around 2.4 GHz. This set of experiments are conducted in indoor environment as shown in Figure 1c. (a) Real parts of time-domain down-converted signals where 18 random control patterns of metasurface are considered. This figure clearly shows very serious in-band and out-of-band disturbances. (b) Real parts of 18 denoised time-domain signals in (a) through the proposed Filter-CNN. (c) Real parts of mean-valued-filtered signals of 2b. (d)–(f) are the spectrum amplitudes corresponding to those in (a)–(c). (g) 18 random control patterns of the metasurface used in this set of experiments.

**Figure 3:**
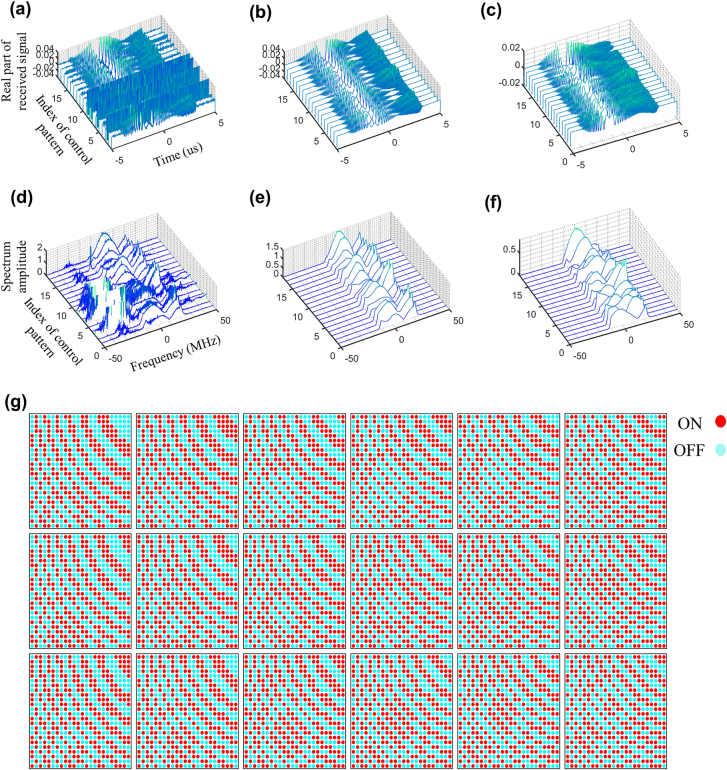
Experimental results of the signal denoise for proposed intelligent EM metasurface camera working at around 2.4 GHz in outdoor environment. (a) Real parts of time-domain down-converted signals where 18 focusing control patterns of metasurface are considered. (b) Real parts of 18 denoised time-domain signals in (a) through the proposed Filter-CNN. (c) Real parts of mean-valued-filtered signals of (b). (d)–(f) are the spectrum amplitudes corresponding to those in (a)–(c). (g) 18 focusing control patterns of the metasurface used in this set of outdoor experiments.

Figure 2b and e presents the results for the denoised signals, where 18 random control coding patterns of programmable metasurface are used, and a human target stands quietly in indoor environment. For comparison, corresponding down-converted signals are also provided in Figure 2a and d. It can be clearly observed from Figure 2a, b and d, e that the overwhelming unwanted inferences can be well filtered out using our Filter-CNN. Recall Eq. (4), the first term characterizes the direct arrival from the source to receiver, and thus is out of control of the programmable metasurface. To demonstrate the role of the metasurface on the compressive measurements, the first term in Eq. (4) is removed by mean-value filter with respect to the slow time (i.e., measurement index), and corresponding results are plotted in Figure 2c and f. From these figures, we see that the programmable metasurface can flexibly manipulate the acquisition of the microwave signals carrying the target’s information, implying that the target’s information can be efficiently captured by a fixed receiver in a compressive way. Additionally, 18 random control coding patterns of the metasurface involved in Figures 2a–f have been plotted in Figure 2g. Recall Eq. (2), one interesting conclusion can be observed, i.e., the good measurements imply the acquired signals with good signal-to-noise (SNR), which can be achieved by controlling the coding patterns of the programmable metasurface. Intuitively, such measurement strategy can be conceived by designing the control pattern of the metasurface such that the resultant radiation beams are focused toward the target, where the prior on the target’s location can be estimated from the image obtained at last time. Results with focusing measurement strategy, corresponding to Figure 2, are presented in Figure 3, from which we can draw two conclusions, i.e., (i) above conclusions can be observed again, and (ii) the SNRs of acquired radio signals can be remarkably improved by using the focusing measurement strategy.

### 4.2 *In-situ* sensing results

We here examine experimentally the performance of the developed metasurface camera for the *in-situ* imaging of human freely acting in our lab. One critical issue to the developed EM metasurface camera is to train it in a supervised way. For this purpose, a commercial optical binocular camera ZED2 from Stereolabs [37] is integrated and synchronized with the EM metasurface camera through the host computer. The optical videos by the ZED2 are utilized as the labeled training samples after a sequence of processes including background removal, segmentation and IUV-transformation through Densepose [38]. In our experiments, our EM metasurface camera is trained by inviting one person (Zhuo Wang, the 1st author of this paper, referred to as training person) acting freely in our lab, and tested by another person (Hongrui Zhang, the 2nd author, referred to as test person). We have collected $8\times 10^{4}$ pairs of labeled training videos, and take around 18 h to train our EM camera. The proposed EM metasurface camera, once being well trained, can produce a high-fidelity videos of test person with the frame rate of about 20 Hz.

**Figure 4:**
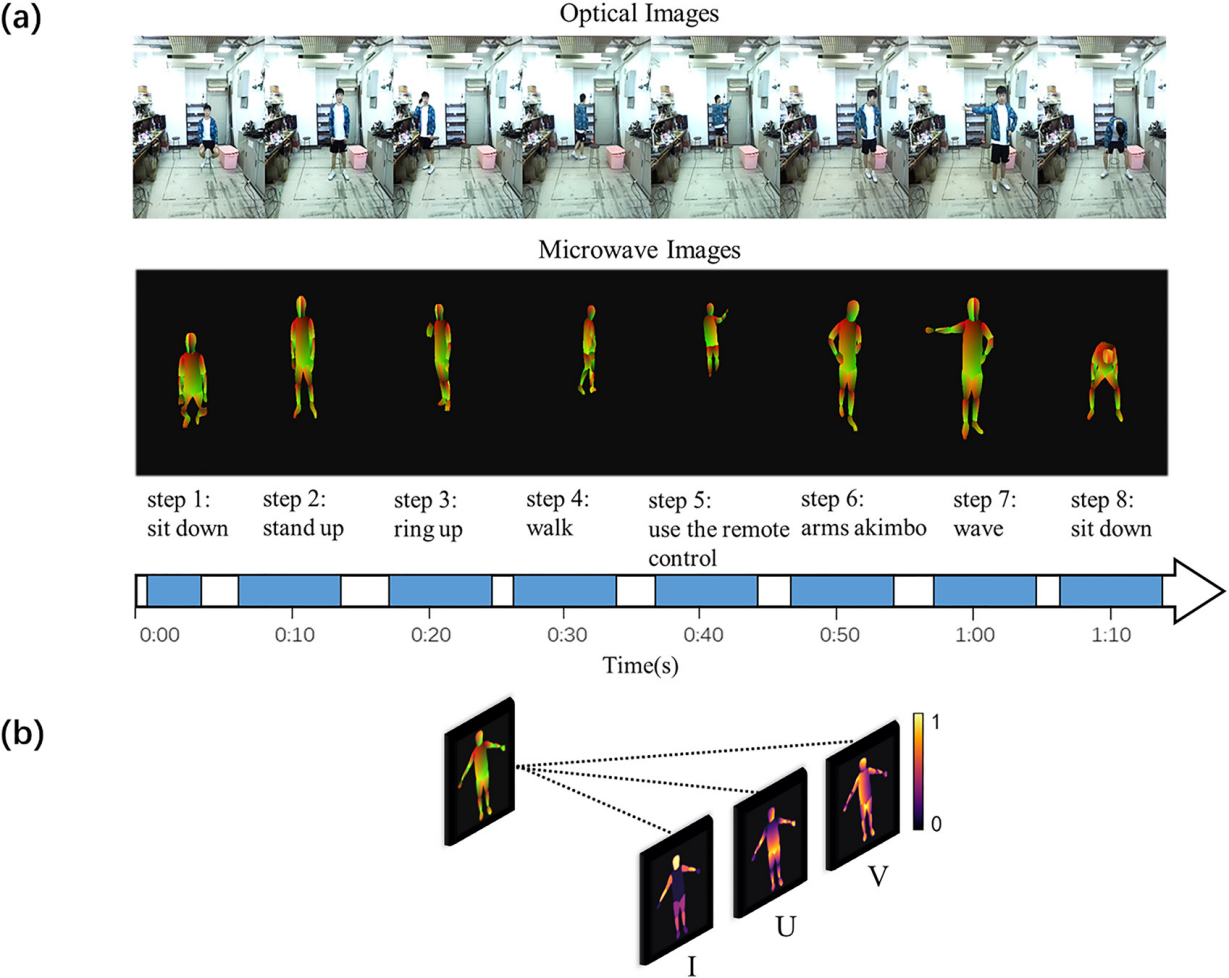
Experimental imaging results in indoor environment and the description of IUV image composition. (a) Experimental *in-situ* imaging results of the test person freely acting in indoor environment shown in Figure 1d. (**Top**) The optical RGB images at selected moments, which are recorded by optical ZED2 camera. (**Middle**) The IUV images recorded by our intelligent EM metasurface camera at selected moments corresponding to those in top row. (**Bottom**) Time line. (b) An IUV image has three channels: I-channel, U-channel, and V-channel. The I-channel image is the classification of pixels that belong to either background or different parts of body, which provide a coarse estimate of surface coordinates. The UV channels provide the result of mapping all human pixels of an RGB image to the 3D surface of the human body.

**Figure 5:**
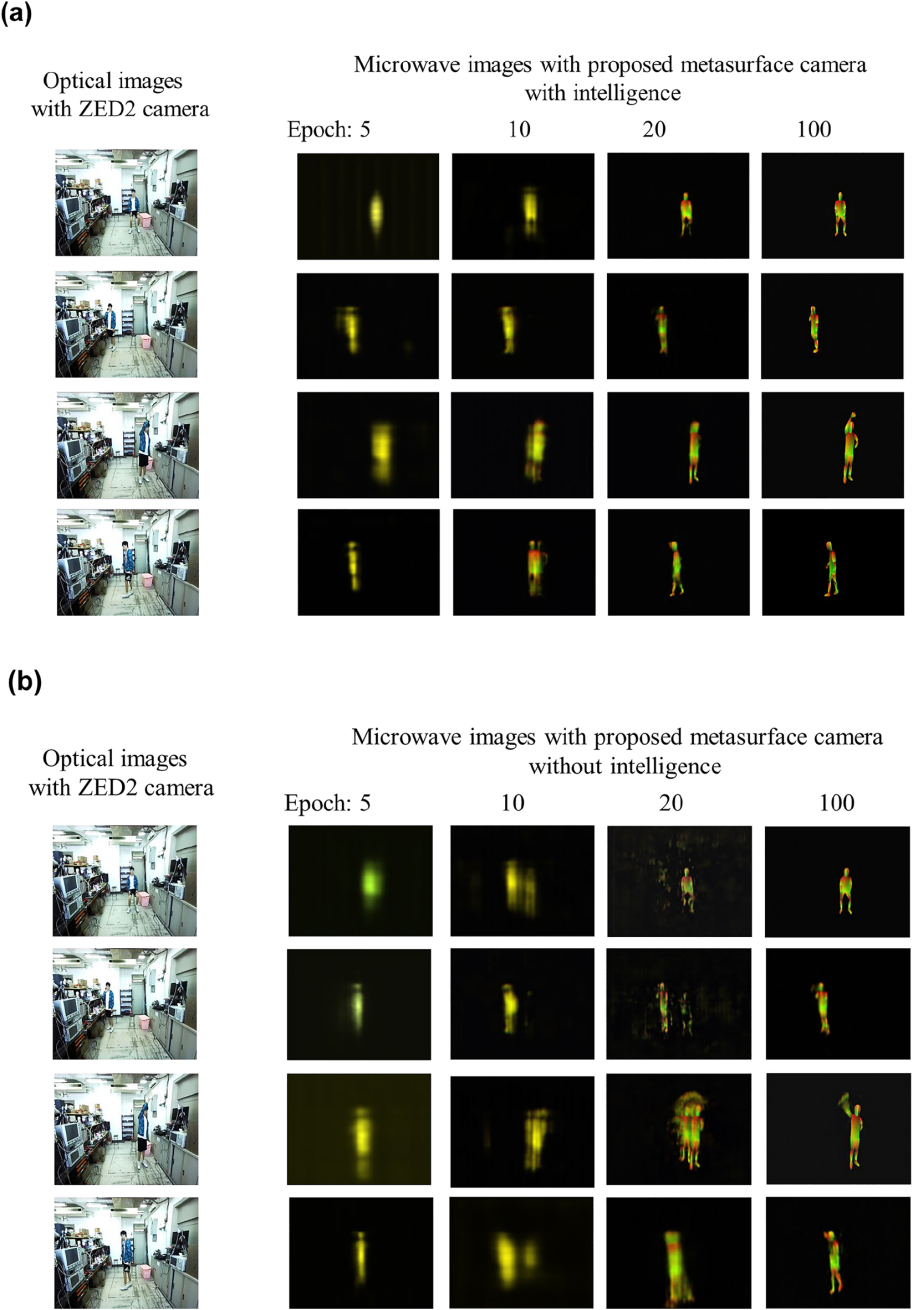

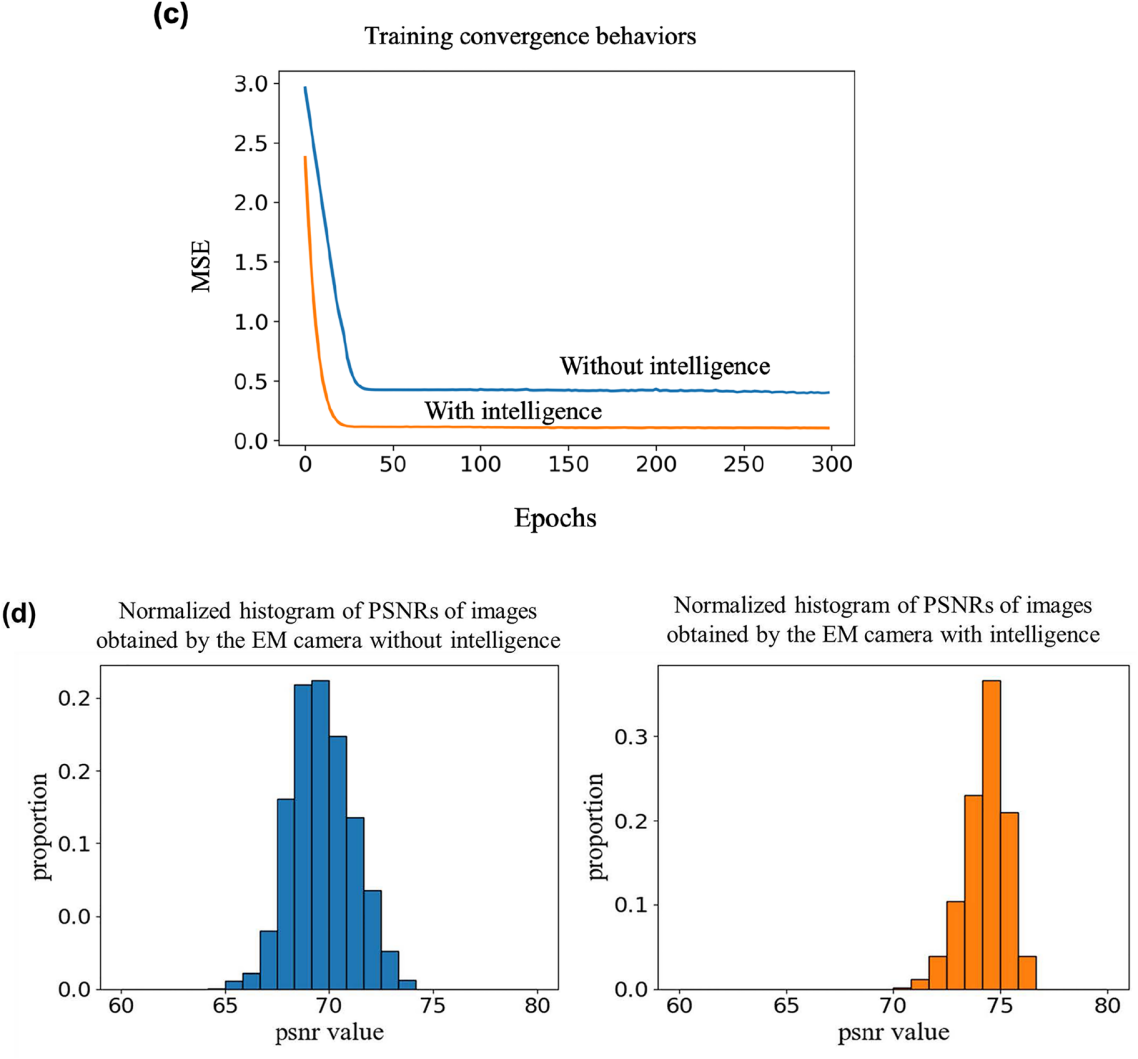
Performance evaluation of proposed EM metasurface camera for sensing human behaviors in indoor environment. (a) and (b) correspond to selected IUV images of test person with four different gestures with varying training epochs, where the intelligence is involved and not, respectively. (c) Comparison of convergence behaviors of MSEs over 1000 1.2 s-length microwave videos as function of training epochs when the intelligence is involved (blue line) or not (orange line). (d) Figures in Left and Right are for the normalized histograms of PSNRS over 1000 1.2 s-length microwave videos recorded using our EM metasurface camera with or without intelligence.

Figure 4 reports a sequence of IUV microwave images at several selected moments from a video recorded by proposed EM metasurface camera, from which one can readily recognize the actions of the test person in indoor environment, for instance, sitting down, fighting, making phone calls, walking, turning on the air conditioner, standing with arms akimbo, waving hands, and so on. As discussed in Section II, the proposed EM camera has the property of intelligence enabled by the adaptive data acquisition and processing. To show the advantage benefited from the intelligence, we conduct a set of experiments and plot the results in Figures 5a–d. Figure 5a presents the selected IUV microwave images of test person with four different gestures with growth of training epochs, where the intelligence is involved. For comparison, corresponding results without the intelligence are also provided in Figure 5b. It can be visually observed that the proposed EM metasurface camera with the intelligence has the faster learning ability than that without intelligence, and that it, once being well trained, exhibits good sensing performance regardless of the use of intelligence. To evaluate quantitatively the performance of proposed EM camera, we make examinations in terms of the training convergence curve and the quality of test images. Figure 5c compares the training convergences when the intelligence is used or not, which shows that the intelligence is helpful in improving the training efficiency. Specifically, it is enough to arrive at the convergence state using around 10 epochs when the intelligence is involved; however, more training epochs are needed. Of course, readers can notice from Figure 5c that the intelligence has smaller loss error than that without intelligence. Figure 5d compares quantitatively the PSNR’s histogram (here, PSNR denotes the peak SNR index) of the test results when the intelligence is involved or not, which shows the benefit of the intelligence.

**Figure 6:**
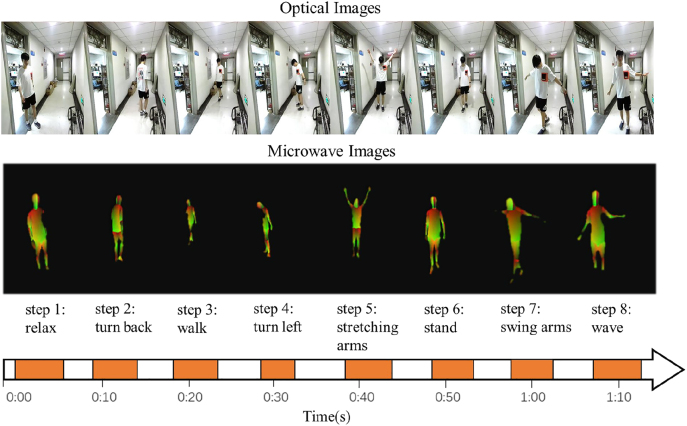
Experimental *in-situ* imaging results of the test person freely acting in a corridor outside our lab with a 60 cm-thickness load-bearing concrete wall. (**Top**) RGB images recorded by optical ZED2 camera at selected moments. (**Middle**) IUV images by our intelligent EM metasurface camera at selected moments corresponding to that in top row. (**Bottom**) Time line.

Now, we examine the through-wall sensing performance of developed EM metasurface camera. In this setting, the target freely acts in corridor outside our lab with a 60 cm-thickness load-bearing concrete wall. The training and test procedures are the same as those in indoor case. Figure 6 reports through-wall IUV images at selected moments recorded by our intelligent EM metasurface camera. It can be observed from this set of figures that the image quality in outdoor environment is comparable to those in indoor environment, and that the actions of the test person behind a 60 cm-thickness concrete wall remains to be clearly identified. To further demonstrate the benefit enabled by the intelligence, we have conducted a set of experiments with random control coding patterns of metasurface, and no acceptable results have been obtained due to the really weak measurements. Therefore, we conclude that the proposed EM metasurface camera with the intelligence has better learning ability than that without the intelligence.

**Figure 7:**
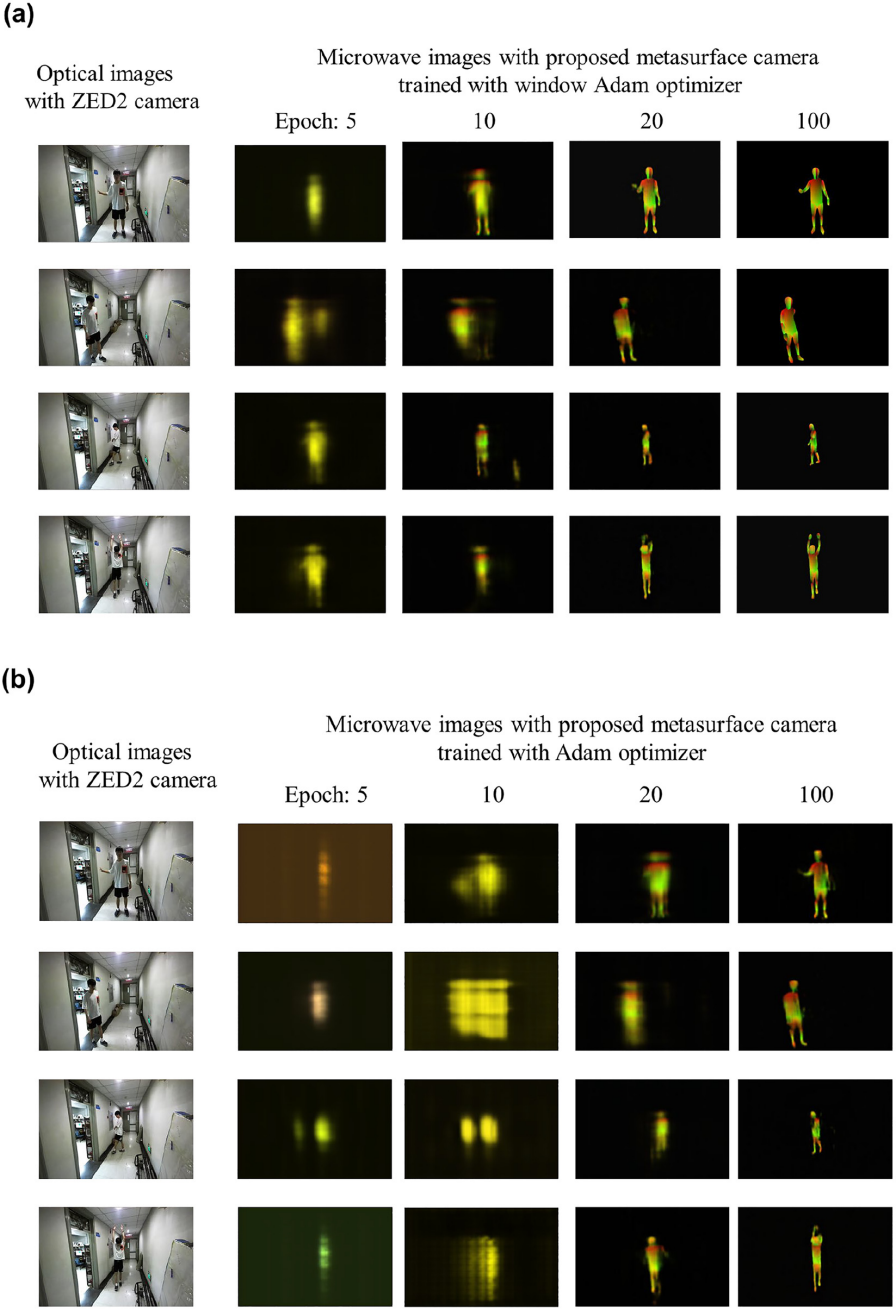

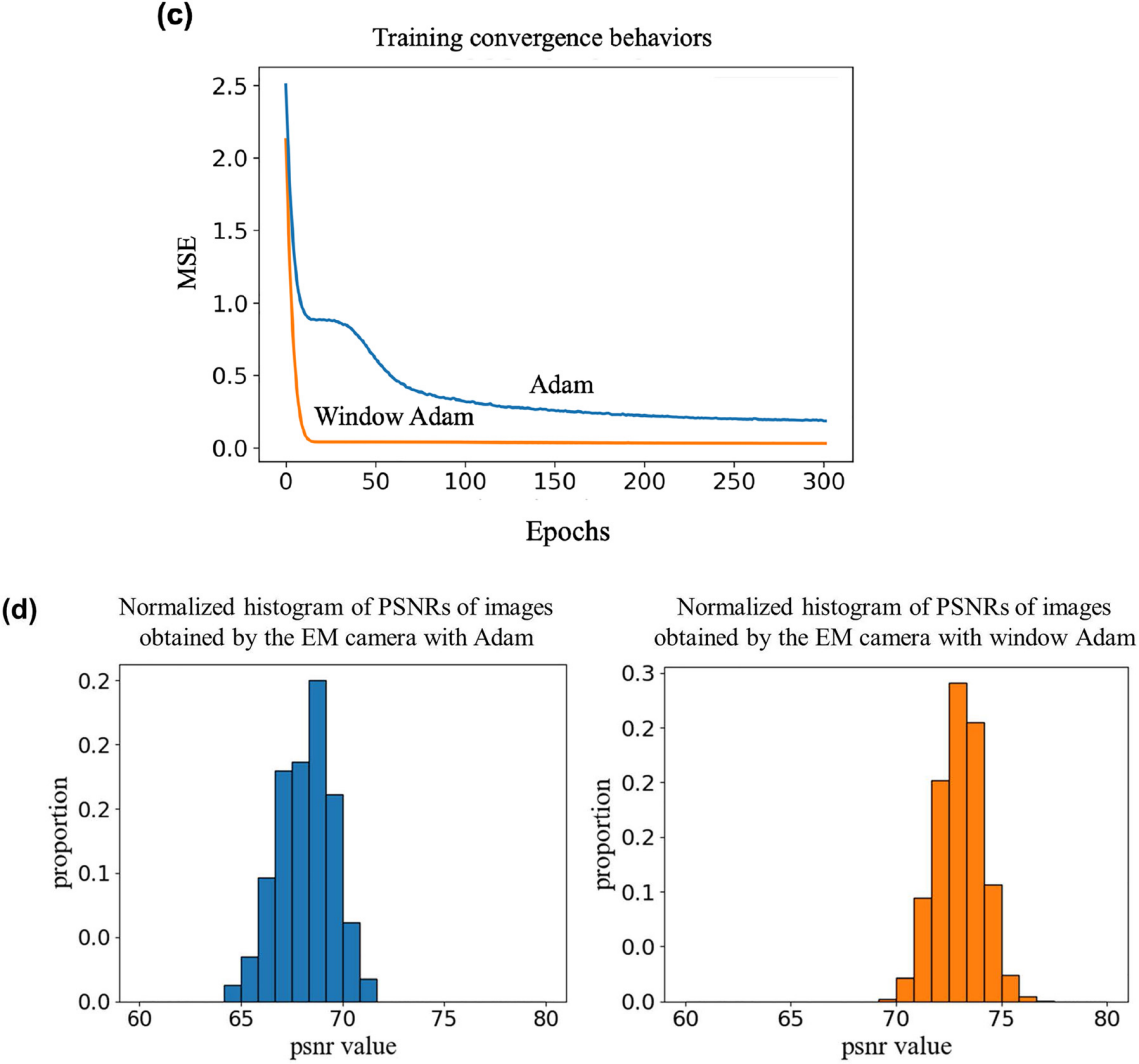
Performance evaluation of our intelligent EM camera for sensing human behaviors in through-wall environment. (a) and (b) correspond to selected IUV images of test person with four different gestures with varying training epochs, where the window-Adam and Adam are used, respectively. (c) Comparison of convergence behaviors of MSEs over 1000 1.2 s-length microwave videos as function of training epochs when the window-Adam (orange line) and Adam (blue line) optimizers are used, respectively. (d) Figures in Left and Right are for the normalized histograms of PSNRS over 1000 1.2 s-length microwave videos recorded by using our EM camera trained with the Adam and window Adam optimizer, respectively.

Before concluding this part, we would like to consider the effect on sensing performance from the use of window Adam optimizer, where the adaptive focusing measurement is considered. Figure 7a and b presents the IUV images of test person with four different gestures with growth of epochs when the Adam and window Adam optimizers are used, respectively. Figure 7c compares the training convergences when the window operation in Adam optimizer is used or not. Figure 7d compares quantitatively the PSNR’s histogram of test results, showing the benefits of the window Adam optimizer to conventional Adam optimizer. From above results, several conclusions can be drawn. First, the window operation in Adam optimizer is really helpful in improving the training efficiency. Notably, during the training process, the location knowledge of target can be rapidly learned by our EM camera, meanwhile reducing ghosting, blurring and other can be reaped. Second, the proposed intelligent EM camera enables us to see clearly human behaviors in a complicated visually-invisible environment.

## 5 Conclusions

In this work, we cast the concept of intelligent EM metasurface camera by deploying the inexpensive programmable metasurfaces along with deep learning strategies, and then establish a Bayesian inference framework for it. We discuss how programmable metasurface and deep learning methods can play a compelling role across the entire sensing chain. The developed intelligent EM metasurface camera relies on two critical issues: (1) a large-aperture programmable metasurface for adaptive data acquisition; and (2) artificial neural networks for instant data processing. We build a proof-of-principle prototype system of intelligent EM metasurface camera working at around 2.4 GHz, and demonstrate experimentally that the EM metasurface camera is capable of enabling us to see clearly human behaviors behind a 60 cm-thickness concrete wall, showing its robust performance in remotely monitoring notable human behaviors in real-world settings. Again, we would like to highlight that the frame rate can be optimized to be in order of tens of KHz in principle if more specialized transceivers are used. The presented strategy opens up a promising route toward smart community, internet-of-things (IoT) connectivity and beyond, and can be transposed to other frequencies and other types of wave phenomena.

## Data availability

The data that support the findings of this study are available from the last author upon request.

## Code availability

Code that supports the findings of this study is available upon reasonable request from the last author.
